# Case of Hœmoptysis, in Which a Very Large Quantity of Blood Was Lost

**Published:** 1828-07

**Authors:** T. Chevalier


### HÆMOPTYSIS.

*Case of Hæmoptysis, in which a very large quantity of Blood was lost.* From the posthumous Papers of the late T. Chevalier, Esq. F.R.S. &c. &c.

Mr. G., ætat. sixty, a stout plethoric person, weighing about sixteen stone, was awaked early in the morning of the 25th of October, by a cough accompanied with expectoration of blood.

Mr. Morley arrived within twenty minutes, and found the heart and arteries acting with great violence. Blood was drawn from the arm until the impetus of the circulation was allayed, and the hæmoptysis ceased; when it was estimated that the patient had lost about ten ounces of blood from the lungs, and twenty-four from the arm. The following draught was ordered to be taken every third hour:

℞. Inf. Rosæ f℥jss.; Acidi Sulph. dil., Tr. Digitalis āā m. xv. M.

At noon, Mr. G. had the benefit of Mr. Chevalier's advice. The hemorrhage had not recurred, but the pulse had resumed its bounding character.

V.S. ad ℥xvj.—℞. Plumbi Superacetatis gr. jss.; Aceti distill. f℥j.; Mist. Amygdalarum f℥j.; Aq. Pimentæ f℥ij.; Tr. Sennæ f℥jss. M. fiat haustus sextâ quâque horâ sumendus.

In the evening, the pulse acquired its former hardness, and the patient complained of costiveness and pain in the bowels.

V.S. ad ℥xvj.—℞. Infus. Sennæ f℥xj.; Tr. Ejusdem f℥j.; Magnes. Sulph. ʒ iij. M. fiat haustus quamprimum sumendus.

26th.—Mr. G. passed a quiet night; during the day also he was free from hemorrhage, and the arterial action appeared much diminished. The bowels slightly relieved.

℞. Potassæ Subcarb., Acidi Citrici, Pulv. Tragacanth. c. āā ℈j.; Potassæ Sulph. ʒ ss.; Aquæ puræ f℥ xij. M. fiat haustus sextâ quâque horâ sumendus.

27th.—At three o'clock in the morning the hemorrhage returned, and nine or ten ounces of blood were expectorated. The force of the circulation was much augmented.

V.S. ad ℥xvj.—℞. Plumbi Superacet. gr. jss.; Aceti distill. fʒj.; Syrupi fʒj.; Aquæ Menth. pip. f ʒ v. M. fiat haustus sextâ quâque horâ sumendus.

In the evening the hemorrhage returned, but was immediately arrested by

V.S. ad ℥xvj. Rep[r] Haustus Sedativus.

28th.—In the morning, Mr. G. complained of pain in the bowels and of constipation. No hemorrhage had taken place during the night. The pulse was less bounding.

In the evening, the arterial action increased.

V.S. ad ℥xvj.

29th.—At five o'clock in the morning, the hemorrhage returned with as much violence as on the first occasion, and from fourteen to sixteen ounces of blood were expectorated.

V.S. ad ℥xxiv.

30th.—The disposition to hæmoptysis appeared much reduced; but the patient complained of considerable abdominal pains and costiveness.

℞. Pulv. Digitalis gr. j.; Conf. Opii gr. iij. M. fiat pilula sextâ quâque horâ sumenda.—℞. Inf. Sennæ f℥ss.; Mist. Camphoræ f℥viij. M. fiat haustus sextâ quâque horâ sumendus.

In the evening, the pulse having reacquired a great degree of hardness,

Appl^r nuchæ Cucurb. Cruentæ, et detrah^r sang. ad ℥xvj.

31st.—Mr. G. complained again of abdominal pains, without tenderness: the bowels continuing costive.

Enema Sennæ cum Magn. Sulph. statim.—℞. Magnes. Sulphatis ℥j.; Aquæ Menthæ f℥vj. M. Capiat quartam partem quartâ quâque horâ.

November 1st.—At five o'clock this morning, the pains in the abdomen continuing, hot fomentations were applied, with considerable relief; emollient clysters were exhibited, and the bowels freely evacuated. At eight o'clock the hæmoptysis returned with increased violence, producing sixteen ounces of blood from the lungs. It lasted until half-past nine: during its continuance, Mr. G. was twice bled from the arm, viz.

V.S. ad ℥xij. et V.S. ad ℥x.—℞. Ol. Terebinth. rect. gtt. xv.; Mucil. Acaciæ, Tr. Kino āā f℥j.; Aq. Rosæ f℥x. M. fiat haustus sextâ quâque horâ sumendus.

At four o'clock, Mr. G. again expectorated blood to the extent of six ounces, the heart beating with great violence.

R. Potassæ Nitr. ℈ss.; Vini Ipecac. f℥j.; Tr. Digitalis m. xv.; Aquæ f℥xj. M. fiat haustus tertiâ quâque horâ sumendus.

In the evening, the patient was relieved; but, the bowels being uneasy, the opening draughts were resumed.

2d.—Mr. G. slept during several hours in the night; the pulse this morning gained strength.

Rep^r Haust. Terebinth.

In the evening, it became necessary to repeat the bleeding.

V.S. ad ℥xiv.

3d.—The force of the circulation was much reduced. The abdominal pains had returned, and he complained of costiveness.

Capiat statim Olei Ricini ℥j.—Enema Emolliens.—Rep^r Haustus Terebinthinæ.

4th.—The disposition to hemorrhage still continued to decline; but the abdominal pains were very severe, and the patient complained of difficulty in voiding his urine.

Fotus Papaveris.—Rep^r Haustus Terebinthinæ.

5th.—The hæmoptysis recurred at different periods from twelve until nine o'clock A.M., by which time about twenty ounces of blood were expectorated. Emetics were given repeatedly during this period.

At half-past nine o'clock, the aid of Dr. Cholmondely was requested, who prescribed the following—

℞. Sulph. Zinci, Pulv. Ipecac. āā gr. v.; Aq. Puræ f℥xij. M. fiat haustus tertiâ quâque horâ sumendus.

At eight in the evening, it was agreed that the state of the circulation required V.S. ad ℥xiv.

℞. Zinci Sulphatis gr. ij.; Pulv. Ipecacuanhæ gr. v. M. fiat pulvis quartâ quâque horâ sumendus.—Capiat primo mane Olei Ricini ℥j.

6th.—About eleven o'clock this morning, the hæmoptysis recurred in a slight degree.

℞. Succi Limonis f℥j.; Carb. Sodæ ʒiij.; Aqua puræ f℥ss. M. fiat haustus sextâ quâque horâ sumendus.

In the evening, V.S. ad ℥xiv.

Pulv. Ipecac. comp. ℈j. horâ decubitus sumenda.

7th.—Mr. G. appeared infinitely better: he had slept during the greater part of the night; he was free from pain, and the activity of the heart and arteries was considerably reduced.

Cont^r Haustus Limonis, et Pulv. Ipec. comp. horâ somni.

8th.—The force of the circulation and the throbbing of the heart were again much increased.

V.S. ad ℥xviij.—℞. Inf. Digitalis f℥ss.; Aquæ Menthæ f℥j. M. fiat haustus quartâ quâque horâ sumendus.—Capiat etiam quartâ quâque horâ Extr Humuli gr. v.

9th.—Mr. G. seemed better in every respect.

Cont^r Haustus et Pilula.

10th.—He complained of considerable pain in the abdomen, the bowels not having been relieved since yesterday morning.

Enema Sennæ quamprimum.—℞. Inf. Digitalis, Aquæ Menth. āā fʒvj. M. fiat haustus sextâ quâque horâ sumendus.

In the evening, the pulse becoming full, and the hæmoptysis returning, V.S. ad ℥x.

℞. Hydr. Submur. gr. iv.; Pulv. Ipecac. comp. ℈j. M. fiat pulvis horâ somni sumendus.—Capiat primo mane Ol. Ricini ℥j.

11th.—About three o'clock this morning, the expectoration of blood recurred, but ceased when ten ounces of blood had been taken from the arm.

℞. Succi Limonis f℥j.; Sodæ Carbon. ʒiij.; Inf. Digitalis f℥ss. M. fiat haustus sextâ quâque horâ sumendus.

In the evening, V.S. ad ℥xvj.

Rep^r Pulvis Ipec. comp. cum Calomel.

12th.—Today, for the first time, the character of the pulse became sensibly altered: the blood appeared to circulate without any hardness of pulsation in the arteries. In the night, the hemorrhage returned, but it was suppressed by V.S. ad ℥vj.

Rep^r Haustus et Pulvis.

From this period Mr. G. progressively improved. His mouth became affected by the Calomel on the 15th. The bowels, by the occasional use of castor-oil, afforded frequent evacuations of dark-coloured fæces, and the urine was abundantly secreted. From the commencement of the attack until now, there never were any febrile symptoms, nor did the constitution of the patient appear to suffer, on the other hand, from the loss of so large a quantity of blood. The frequency of the pulse may be averaged at 72, varying from 60 to 86.

The total loss of blood between the 25th of October and 12th of November, Mr. Morley estimated as follows: From the lungs, 87; from the arm, 254; by cupping, 16; total, 357 ounces.

P.S.—It is now about seven years since the date of this case, and the patient has been dead about three months.

